# Molecular Profiling in Resectable Colorectal Liver Metastases: The Role of KRAS Mutation Status in Assessing Prognosis in the Preoperative Setting

**DOI:** 10.6004/jadpro.2015.6.5.7

**Published:** 2015-09-01

**Authors:** Ryanne Coulson

**Affiliations:** MD Anderson Cancer Center, Houston, Texas

Molecular biomarkers are increasingly being utilized as both prognostic and predictive tools in the care of patients with cancer. Testing for alterations in biomarkers is currently considered the standard of care in a growing number of cancers, including breast, lung, leukemia, and colorectal. Recently updated National Comprehensive Cancer Network (NCCN) guidelines recommend testing for alterations in *KRAS*, *NRAS*, and *BRAF* as part of the initial workup for metastatic colorectal cancer (CRC; NCCN, 2015).

The presence of *KRAS* mutation occurs in 35% to 45% of metastatic CRC (Andreyev et al., 2001; Andreyev, Norman, Cunningham, Oates, & Clarke, 1998; Lievre et al., 2006; Neumann, Zeindl-Eberhart, Kirchner, & Jung, 2009; Richman et al., 2009) and has been shown to be associated with a worse prognosis in both metastatic and high-risk stage II/stage III CRC (Andreyev et al., 2001; Andreyev et al., 1998).

The presence of *BRAF* mutation occurs in about 10% of patients with metastatic CRC and is associated with a poor prognosis (Tie et al., 2011; Tran et al., 2011). The study by Karagkounis et al. (2013), which is discussed by Wei and Samp on page 460, looked at the incidence and prognostic significance of *KRAS* (codons 12, 13) and *BRAF* *V600E* mutations in patients with metastatic CRC undergoing liver metastasectomy. This study found that *KRAS* mutation was an independent predictor of overall and recurrence-free survival following liver surgery for colorectal metastases.

## Study Strengths

The study by Karagkounis and colleagues (2013) evaluated the outcomes following liver metastasectomy in patients with metastatic CRC at Johns Hopkins Hospital over a 5-year period in relation to molecular mutations in *KRAS* and *BRAF*. The clear strengths of this study include its size (N = 202) and the complexity of the representative population.

Of this group of patients, 81% received preoperative chemotherapy, 33% had three or more metastases, 64% had synchronous disease, and 25% were treated with a combination of resection and ablation. These independent factors of prognosis were analyzed, and *KRAS* mutation remained predictive for overall and recurrence-free survival. By including patients who received chemotherapy, had bilobar disease, had an intact primary tumor, and were treated with surgery plus ablation, the study was representative of the complex patients often seen at tertiary institutions.

Additionally, in this study, the surgical and ablative therapies were performed at a single institution, which provides a consistent quality of technique and minimizes the impact of surgical quality on the analysis of other independent factors. Additionally, testing for *KRAS* and *BRAF* mutations was performed by the same pathology department utilizing a single methodology, which allowed for consistent, reliable results in molecular biomarker testing.

Another advantage of the study was the analysis of the impact of *KRAS* and *BRAF* mutations on overall survival after hepatic surgery for CRC metastases by both univariate and multivariate analyses. This analysis provides clinicians and patients with important information about risk and expected outcomes from liver surgery in metastatic CRC in relation to alterations in molecular biomarkers.

**Figure 1:**
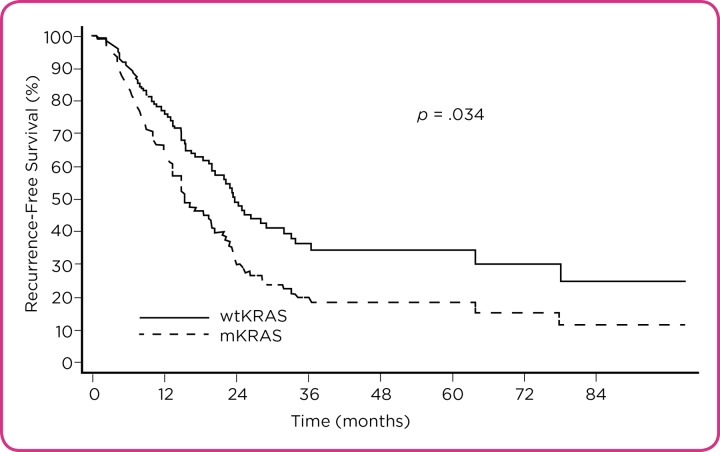
Recurrence-free survival after hepatic surgery for colorectal liver metastasis depicted by KRAS mutation status (multivariate Cox model). wtKRAS = wild-type KRAS; mKRAS = mutant KRAS. Reprinted from Karagkounis et al. (2013) with permission from Wiley

**Figure 2:**
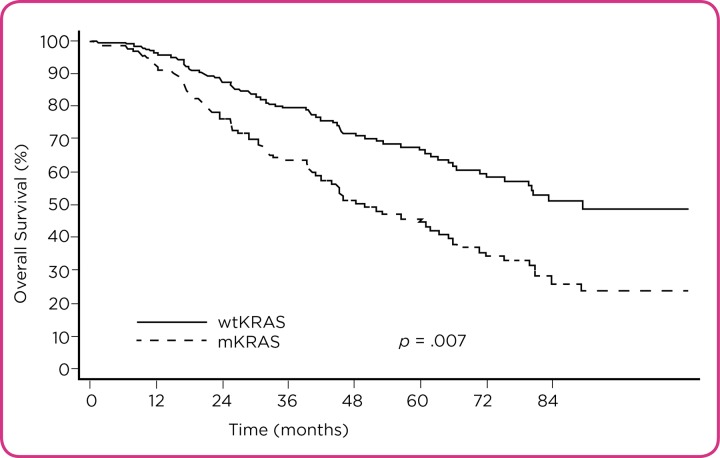
Overall survival after hepatic surgery for colorectal liver metastasis depicted by KRAS mutation status (multivariate Cox model). wtKRAS = wild-type KRAS; mKRAS = mutant KRAS. Reprinted from Karagkounis et al. (2013) with permission from Wiley.

The impact of *KRAS* mutation both on recurrence-free (RFS) and overall survival (OS) following hepatic surgery for CRC liver metastasis is clearly demonstrated using a multivariate Cox model (Figures 1 and 2). The hazard ratio (HR) significantly increases in multivariate analysis compared to univariate analysis and helps to better demonstrate the negative impact of *KRAS* mutation as an independent predictor of OS.

## Use of the Hazard Ratio

The hazard ratio is an estimate of the ratio of the hazard rate in one group vs. a second group. The hazard rate is the probability that an event in question will occur within a given time interval. A hazard ratio of 1 would indicate no difference between the two groups at a given time point.

In clinical trials, the hazard ratio is often used to compare a treatment group vs. a control group, with the endpoint being symptom or disease resolution. For such trials, the hazard ratio indicates the probability of symptom or disease resolution in the treated vs. control subjects at any given time point (Spruance, Reid, Grace, & Samore, 2004).

However, in cancer research, the hazard ratio is used slightly differently. The hazard ratio is often used in cancer clinical trials to measure survival at any point in time in a group of patients compared with a second group of patients. A hazard ratio of less than or greater than 1 indicates that survival was better in one group (National Cancer Institute, 2015). In this study of patients who underwent liver surgery for metastatic CRC, *KRAS* mutation was associated with significantly worse OS (HR = 1.99) and RFS (HR = 1.68).

## Summary of Study Findings

A representative population of 202 patients with metastatic CRC were treated with surgical intervention, with 152 undergoing liver resection alone and 50 treated with a combination of surgery and ablation. The median follow-up was 1.5 years and 2.7 years for survivors.

The presence of *KRAS* mutation was detected in 58 tumor samples (29%), and *BRAF* mutation was detected in 4 tumor samples (2%). The presence of *KRAS* mutation was associated with an increase in mortality in univariate analysis (HR = 1.34; 95% confidence interval (CI), 0.88–2.04), but it was not statistically significant (*p* = .173). After adjusting for known predictors of survival, multivariate analysis showed that *KRAS* mutation was associated with significantly worse OS (45.2 months vs. 71.9 months in *KRAS* wild-type) following liver surgery for metastatic CRC (HR = 1.99; 95% CI, 1.21–3.26; *p* = .007).

Subgroup analysis showed a more pronounced decrease in OS in patients with *KRAS* mutation who underwent liver resection and ablation (univariate HR = 2.55; 95% CI, 1.25–5.20; multivariate HR = 7.13; 95% CI, 2.85–17.85). Due to the low incidence of *BRAF* mutations observed in the trial, determining their effect on survival was not possible. However, the few subjects with *BRAF* mutation had poorer OS than those with wild-type *BRAF* (median, 25.4 months vs. 70.7 months).

The median time to recurrence for all subjects was 18.9 months, with a 3-year RFS of 32.2%. The presence of *KRAS* mutation was associated with decreased RFS (univariate HR = 1.53; 95% CI, 0.99–2.36; multivariate HR = 1.68; 95% CI, 1.04–2.70), with a median RFS of 11.8 months in patients with *KRAS* mutation and 20.8 months in patients with wild-type *KRAS*.

## Study Limitations

The main limitations of this study include the limited RAS testing performed and the underrepresented proportion of *KRAS* and *BRAF* mutations compared with the general metastatic CRC population. Karagkounis et al. (2013) evaluated KRAS mutations in codons 12 and 13, which correlated with prior NCCN guidelines. However, patients with *KRAS* mutations in codons 61 and 146 also have shorter disease-free survival and are resistant to epidermal growth factor receptor (EGFR) inhibitor therapy (Loupakis et al., 2009), findings that are similarly observed in patients with *NRAS* mutations (De Roock et al., 2010). Updated NCCN guidelines recommend testing for *KRAS* mutation (both exon 2 and nonexon 2) and *NRAS* mutation during the initial workup of metastatic CRC (NCCN, 2015).

**Figure 3:**
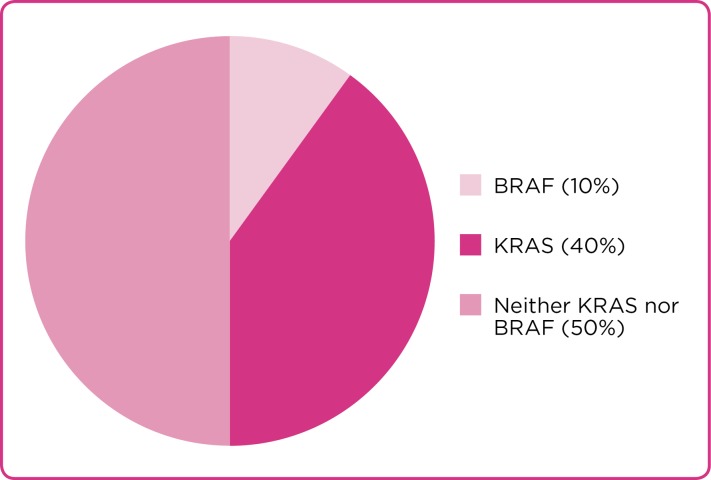
Frequency of KRAS and BRAF mutation rates observed in metastatic colorectal cancer.

**Figure 4:**
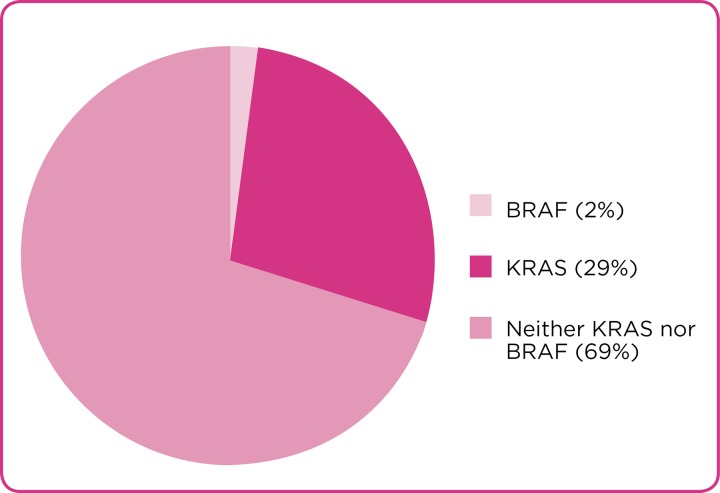
Frequency of KRAS and BRAF mutations observed in the Karagkounis et al. study (2013)

The previously reported incidence of molecular alterations in *KRAS* and *BRAF* was observed at a lower rate in this study (40% vs 29%; 10% vs. 2%, respectively; Figures 3 and 4). This finding may be attributable to the poorer prognoses associated with the mutations, as patients with these mutations may have underlying disease biology that makes them less likely to be surgical candidates. In particular, BRAF mutation in metastatic CRC has been shown to be associated with higher rates of metastases to the distant lymph nodes and peritoneum and lower rates of metastases to the liver and lungs than *BRAF* wild-type tumors (Tran et al., 2011).

Additionally, patients with metastatic CRC with *BRAF* mutation less often have liver-limited disease and have been shown to have shorter survival following metastasectomy than their *BRAF* wild-type counterparts (Yaeger et al., 2014). However, the lower rates of these mutations seen in this study may limit some ability for interpretation and do not allow for meaningful analysis of patients with metastatic CRC who have *BRAF* mutation undergoing liver surgery.

## Practical Implications

Molecular alterations in cancer biomarkers can play a major role in prognosis and treatment options for patients with cancer. More investigation is needed to understand whether selecting surgical options should be influenced by molecular alterations. At present, *KRAS*, *NRAS*, and *BRAF* mutations are associated with poorer outcomes in metastatic CRC, both in surgical and nonsurgical patients.

Physicians and advanced practitioners (APs) should be knowledgeable in this advancing aspect of cancer care to help patients understand the implications of these alterations and how such alterations can affect treatment options, survival, and disease course. Patients who are properly educated about their disease, risk, and treatment options can make better informed decisions regarding their care, ones that are aligned with realistic treatment goals.

## Conclusion

This study provides support for consideration of the role of biomarkers in risk stratification in the surgical treatment of colorectal liver metastases. By allowing for prior chemotherapy, bilobar disease, an intact primary tumor, and treatment with surgery plus ablation, the study by Karagkounis and colleagues (2013) was representative of the complicated and complex patients often seen at tertiary institutions and more accurately demonstrates that *KRAS* was an independent predictor in a truly representative population. The continued advances in tumor profiling and cancer genetics allow for increasingly personalized cancer therapy. Understanding how these advancements affect individual patients and educating them on their disease and treatment options remain key opportunities for APs to incorporate this growing knowledge into everyday patient care.
